# Naringenin protects AlCl_3_/D-galactose induced neurotoxicity in rat model of AD via attenuation of acetylcholinesterase levels and inhibition of oxidative stress

**DOI:** 10.1371/journal.pone.0227631

**Published:** 2020-01-16

**Authors:** Saida Haider, Laraib Liaquat, Saara Ahmad, Zehra Batool, Rafat Ali Siddiqui, Saiqa Tabassum, Sidrah Shahzad, Sahar Rafiq, Narjis Naz

**Affiliations:** 1 Neurochemistry and Biochemical Neuropharmacology Research Unit, Department of Biochemistry, University of Karachi, Karachi, Pakistan; 2 Department of Biological and Biomedical Sciences, The Aga Khan University, Karachi, Pakistan; 3 Dr. Panjwani Center for Molecular Medicine and Drug Research, International Center for Chemical and Biological Sciences, University of Karachi, Karachi, Pakistan; 4 Nutrition Science and Food Chemistry Laboratory, Agricultural Research Station, Virginia State University, Petersburg, United States of America; 5 Department of Biosciences, Shaheed Zuifiqar Ali Bhutto Institute of Science and Technology, Karachi, Pakistan; 6 Pakistan Navy Medical Training School and College, PNS Shifa, Karachi, Pakistan; 7 Department of Genetics, University of Karachi, Karachi, Pakistan; Weizmann Institute of Science, ISRAEL

## Abstract

Currently prescribed medications for the treatment of Alzheimer’s disease (AD) that are based on acetylcholinesterase inhibition only offer symptomatic relief but do not provide protection against neurodegeneration. There appear to be an intense need for the development of therapeutic strategies that not only improve brain functions but also prevent neurodegeneration. The oxidative stress is one of the main causative factors of AD. Various antioxidants are being investigated to prevent neurodegeneration in AD. The objective of this study was to investigate the neuroprotective effects of naringenin (NAR) against AlCl_3_+D-gal induced AD-like symptoms in an animal model. Rats were orally pre-treated with NAR (50 mg/kg) for two weeks and then exposed to AlCl_3_+D-gal (150 mg/kg + 300 mg/kg) intraperitoneally for one week to develop AD-like symptoms. The standard drug, donepezil (DPZ) was used as a stimulator of cholinergic activity. Our results showed that NAR pre-treatment significantly protected AD-like behavioral disturbances in rats. In DPZ group, rats showed improved cognitive and cholinergic functions but the neuropsychiatric functions were not completely improved and showed marked histopathological alterations. However, NAR not only prevented AlCl_3_+D-gal induced AD-like symptoms but also significantly prevented neuropsychiatric dysfunctions in rats. Results of present study suggest that NAR may play a role in enhancing neuroprotective and cognition functions and it can potentially be considered as a neuroprotective compound for therapeutic management of AD in the future.

## Introduction

Alzheimer’s disease (AD) is a neuronal degenerative disease and is one of the most financially draining diseases to the society [1]. The AD is associated with neurobehavioral [2] and neuropathological hallmarks together with severe cognitive dysfunctions [3]. The disease is characterized by cortical and hippocampal neuronal degeneration [4]. Neuronal degeneration-induced cognitive deficits, and short-term memory (STM) impairment is generally the first clinical sign of AD [5] and studies have reported that such neurodegenerative mechanisms are under the influence of oxidative stress [6, 7]. Neurofibrillary tangles and Aβ plaques are the main pathological features of AD. Aβ plaque is a pathological product formed by activities of β and γ secretase. Clinical and animal studies have extensively described the potential role of Aβ plaques in the occurrence and progression of AD [8]. In addition, accumulation of intracellular τ protein in the form of neurofibrillary tangles is also extensively reported as pathological hallmark in AD and it is suggested that accumulation of plaques and tangles are mainly initiated and expedited by oxidative stress [9]. The destructive free-radical mediated oxidative stress increases with age, with a decline in the efficiency of endogenous antioxidant defense system [10]. If free radicals are not quickly removed, their accumulation may result in cellular senescence [11]. These presumptions demonstrate the effectiveness of antioxidant therapy in particular cases.

Pharmacological or dietary intake of antioxidant is the most efficient way to enhance endogenous antioxidant defense system and to protect the body from destructive effects of oxidative damage [12]. Polyphenolic compounds are abundantly distributed in nature and display potent antioxidant and free radical scavenging properties [13]. Emerging evidences suggest that phytochemicals improve learning, memory, and other general cognitive functions [14]. Flavonoids are the major class of polyphenols and a broad range of experimental data have suggested potential role of flavonoids in improving general cognitive functions and also in the management of neurological disorders including AD [15], Parkinson's disease (PD) [16] and stroke [17]. Flavonoids constitute the major group of polyphenols predominantly present in nature [18]. Flavonoids have beneficial effects on the vascular system and the improved cerebrovascular functions have been strongly linked to enhance cognitive functions and to delay or even to prevent the progression of many age-associated neurodegenerative processes [19]. Flavonoids exhibit neuroprotection against oxidative stress and inhibit Aβ-induced neuronal death. Beneficial effects of flavonoids are attributed to their antioxidant capacity and to their interaction with various signaling pathways that regulate neuronal survival, differentiation, and death [20–22]. Naringenin (NAR) is an aglycone form of naringin widely present in natural products such as citrus fruits, cherries, and tomatoes [23]. Potent antioxidant and metal chelating properties of NAR have been described in previous studies and its consumption has been associated with prevention against various metabolic disorders [24–26].

Although the development of various medications that can help with several symptoms associated with AD including thinking problems, cognitive dysfunctions, difficulties in language and motor skills, still there is no cure of AD. Currently available medication for the treatment of AD including AChE inhibitors and N-methyl-D-aspartate (NMDA) receptor antagonist can only improve the functions of intact neurons but cannot inhibit the ongoing neurodegenerative process leading to neuronal death [27]. Therefore, there is an intense need for developing therapeutic strategy that not only improves brain functions but also prevents neurodegeneration. The objective of the present work was to investigate the potential antioxidant role of NAR in protecting brain dysfunction against AlCl_3_+D-gal induced AD-like symptoms in rats by assessing various behavioral, histopathological, neurochemical and biochemical parameters.

## Material and methods

### Ethical statement

Thirty young adult male albino Wistar rats weighing 150–200 g, purchased from DUHS-Ojha campus. Rats were kept individually in specifically designed plastic cages in an environmentally controlled room with free access to standard rodent diet and tap water. Prior to experiments, animals were subjected to 1 week of acclimation period and to behavioral processes to nullify the psychological affliction of environment for reducing the novelty and handling stress. All animal experiments were approved by the Institutional Advanced Studies and Research Board (ASRB/01769/Sc.) and were done in strict accordance with National Institute of Health Guide for Care and Use of Laboratory Animals (NIH Publication no. 85–23, revised 2011) and the UK Animals (Scientific Procedures) Act 1986.

### Chemical and reagents

Hydrogen peroxide stock (35%) solution, thiobarbituric acid, ferric chloride, trichloroacetic acid, nitro blue tetrazolium, and dithiobisnitrobenzoic acid were purchased from British Drug House (BDH, Dorset, UK). Hydroxylamine hydrochloride, acetylthiocholine, D-gal, aluminum chloride hexahydrate, NAR, DPZ, octyl sodium sulphate (OSS) and all other reagents were purchased from Sigma Chemical Co. (St. Louis, USA). Sunflower oil was purchased from local market. One step RNA reagent, cDNA Synthesis Kit, DreamTaq Green PCR Mastermix were purchased from Bio Basic Canada Inc, Fermentas, USA and ThermoFischer Scientific, USA, respectively.

### Animals

**Fig 1:**
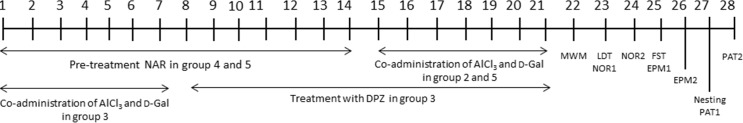
Schematic representation of drug treatment and behavioral assessment. Group specifications are mentioned in Table 1. The days of behavioral tests are expressed along with the abbreviation of test. Abbreviations: MWM–Morris water maze, LDT–Light dark transition, NOR–Novel object recognition, FST–Forced swim test, EPM–Elevated plus maze, PAT–Passive avoidance test.

After screening with Morris water maze (MWM) rats were randomly divided into 5 groups with six rats in each group (S1 Table). Group I assigned as control, Group II assigned as AD-like model, Group III assigned as AD+DPZ, Group IV assigned as NAR and Group V assigned as NAR+AD (Table 1). NAR and DPZ were prepared in sunflower oil and distilled water, respectively. D-gal and AlCl_3_ were prepared in 0.9% saline. This study was divided into two phases. In phase one, rats in Group IV and V were supplemented with NAR (p.o) at a dose of 50 mg/kg for a period of two weeks. Rats in group III were exposed to AlCl_3_ and _D_-gal (i.p) at a dose of 150 mg/kg and 300 mg/kg respectively for 1 week. Control group rats received sunflower oil orally while rats in group II received tap water (p.o). In phase II, after 1 week rats in group III received DPZ (p.o) at a dose of 1.5 mg/kg. After two weeks of Nar pre-treatment, rats in group II and V were then exposed to AlCl_3_ and _D_-gal at a dose of 150 mg/kg and 300 mg/kg respectively for 1 week. Control group rats were injected (i.p) with the same volume of 0.9% saline. All drugs were freshly prepared daily before the start of experiment. Selection of dose and duration was based on our previous studies [28–32]. Twenty-four hours after last dose rats were subjected to behavioral analysis (Fig 1). Rats were sacrificed at the end of last behavior to collect their brain samples. Brains were dissected into hippocampus and cortex. Neurochemical measurements and histopathological studies were done in hippocampus and cortex. Biochemical estimations and DNA fragmentation assay were done in rest of the brain. AChE gene expression was also determined in hippocampal region of rat brain. Each experiment was conducted in a balance design with proper conditions and responses were evaluated in a fixed schedule to avoid time and order effect.

**Table 1:** Drug dose and route of administration of groups.

Groups	N	Treatment	Experimental design
1	6	Control	Sunflower (p.o) + Saline (i.p)
2	6	AD	AlCl_3_ (150 mg/kg) i.p + _D_-gal (300 mg/kg) i.p
3	6	AD+DPZ	AlCl_3_ (150 mg/kg) i.p + _D_-gal (300 mg/kg) i.p + DPZ (1.5 mg/kg) p.o
4	6	NAR	NAR (50 mg/kg) p.o
5	6	NAR+AD	NAR (50 mg/kg) p.o + AlCl_3_ (150 mg/kg) i.p + _D_-gal (300 mg/kg) i.p

Abbreviations: AlCl_3_ –Aluminium chloride, _D_-gal—_D_-galactose, DPZ–Donepezil, NAR–Naringenin, i.p–Intraperitoneally, p.o–Per oral.

### Behavioral analysis

#### Morris water maze

The MWM test is extensively performed to assess the spatial/working memory performance of animals. This apparatus consisted of a circular pool having a diameter of 90 cm and a height of 37 cm. The tank was filled with water (24 ± 2°C) having a depth of 12 cm. Both circular pool and platform were made of white painted metal. The escape platform has a flat metallic top, surface diameter of the top was 8 cm and was placed in the tank 2 cm below the water level. The water within the pool was made opaque by adding powdered milk in order to make the platform invisible and secondly to track the rat's swim paths proficiently. Initially the rat was subjected into the tank to explore it for 2 min. Each rat was then given four consecutive trials to locate the submerged platform in the maze and the interval between each trial was 15 min. Starting positions from arbitrarily assigned compass locations were randomized for each rat on each trial. In the present investigation four starting locations were used: NW, which is the target quadrant and the others were SW, SE, NE. These starting positions were considered so that the animal while using each of the four starting positions was not able to learn a specific order of left or right turns to find the submerged platform. The submerged platform was kept in a constant position throughout all trials during assessment for all the rats. During each trial the time taken by the rat to find hidden platform was noted as escape latency. To evaluate spatial memory after 1 h of learning acquisition a probe trial was performed. During probe trial the platform was removed from its location and the rats were allowed to swim for 120 s, during this time the parameters that were assessed during probe trial included the number of entries over the target quadrant and the time spent in the target quadrant and as an index of reference/ spatial memory [33].

#### Novel object recognition

Novel object recognition (NOR) has been extensively used for determining alterations in cognitive functions as it can measure preference of novelty in rodents. The preference of novel object may indicate the existence of familiar object presentation in memory of a rat. The procedure performed in this study was same as already described previously [28]. In order to monitor recognition memory, rats were exposed to two similar objects at the same time and their discrimination ability was monitored to discriminate between novel and familiar object. The NOR apparatus is made of square wooden box that was painted grey with dimensions of 45 × 45 × 45 cm^3^. Cleaning of box was not allowed throughout the experimental period in order to saturate it with olfactory stimuli. The objects used were two similar transparent glasses (used as familiar objects A1 and A2) and to make them heavy enough the glasses were filled with cement so that rats could not move them from their position. Metallic container (used as novel object B) of same size was also filled with white cement. The size of all objects (novel and familiar) was 2.5 times the size of the rat so that the rat could easily sniff them. The test was divided into three phases: habituation, training, and test session. During habituation the rat was exposed to the box for 10 min. Twenty four after habituation, the rat was again exposed to the box for training phase during which two familiar objects was placed inside the box (A1 and A2) and was given 5 min to explore the objects and the box. Rat was removed after 5 min from the box and was placed back into home cage. Before the start of the test phase 20 min were allowed to elapse. During the test phase, rat was placed into the box again and one familiar object was replaced by novel object (B) and was allowed to explore for 3 min. Novel and familiar object sniffing time was recorded. Exploration of an object was regarded as when the rat directed its nose toward the object at a distance of <2 cm. To assess recognition memory in rats total exploration time of novel and familiar objects were used to calculate discrimination index by using the formula: time spent on novel object−time spent on familiar object)/ (time spent on novel object + time spent on familiar object.

#### Passive avoidance test

The apparatus used for passive avoidance test (PAT) consists of two compartments of equal size, one compartment is dark and it is referred as punishable and the other is light and referred as safe. A door linked both the compartments and it allows the free crossing of rat from one to another compartment. Both compartments contain grid floor that consist of rods, distance between two rods was 0.5 cm and each rod has a diameter of 5 mm. Associative memory was assessed in PAT and it consists of two phases: training and test. For training the rat was placed in light compartment, the moment rat enter the dark compartment with all its four paws, it received a foot shock of 1.5 mA via the rods of grid floor and then rat moved back into the light compartment. STM was assessed after 60 min of training phase. During the test session the rat was placed again into the light compartment and the time rat take to enter into the dark compartment was observed as step through latency. The cut-off time for test phase was 180 s. After 24 h of training phase rats were subjected to test LTM [28].

#### Elevated plus maze

Elevated plus maze (EPM) is a behavioral task to assess working memory in rats. EPM used in the present work consisted of four arms, two open arms (50 × 10 cm) that crossed with two closed arms of equal dimensions. The closed arms have walls of 40 cm. A central square area (10 × 10 cm) connects the four arms and gives maze an appearance of plus sign. The maze was raised above the floor at a height of 60 cm. The procedure and techniques were kept the same as previously described [28]. The memory was assessed in two trials. First is training phase, during which the rat was placed at one end of open arm and the position of each rat was in such a way that its face was opposite to the central square. The transfer latency was noted by using stop watch. Transfer latency is defined as the time rat takes to enter into any of the closed arm with all its four paws. To monitor the retention in memory test phase was conducted 1 h (STM) and 24 h (LTM) after training to monitor working memory performance in terms of transfer latency. The cut-off time which is the time given to each rat to explore the maze was 2 min for both phases. The index for memory impairment is the significant increment in the transfer latency.

#### Nesting behavior

Protocol for nesting behavior was same as described previously [34]. Test for nesting behavior in rats was performed overnight. To assess this behavior rats were placed individual in their home cages with normal bedding covering the floor to a depth of 0.5 cm. A nestlet (square of 2 g pressed cotton) was placed in each cage. Nesting score (0–5) was used to assessed nesting behavior in rats. Nesting score were assigned as; 0 = untouched/ intact nestlet; 1 = largely untouched nestlet (> 90% remains intact); 2 = partially torn up nestlet (50–80% intact); 3 = mostly shredded nestlet (50% of the nestlet remains intact); 4 = flat but an identifiable nest (> 90% of the nestlet is torn up and used); 5 **=** near to perfect nest (used the nest is a crater with walls).

#### Forced swim test

Forced swim test (FST) is pharmacologically recognized and commonly used model for assessing depression in rats. The depression-like behavior in rats is defined as the termination of persistently escape-directed behavior when the rats were placed in an inescapable chamber filled with water. In order to observe depression, rats were forced to swim in a glass tank filled with water. FST apparatus consists of a glass tank the width of the tank was 20 cm and the height was 56 cm. The tank was filled with water (24±2°C) to the height of 22 cm so that the animal was not able to touch the bottom of the tank. Rat’s activity in the glass tank was observed for 300 s in terms of struggling time. The struggling time is defined as the time during which the rat struggles to escape from the tank whereas immobility time is the time when the rat becomes immobile and shows no more efforts to escape [28]. Immobility time was calculated by the following formula: immobility time = [300 s—struggling time].

#### Light/dark transition test

Light/dark transition (LDT) test was used to assess anxiety in rats in present work, the apparatus consists of two compartments one was light compartment made up of transparent plastic and the other was dark compartment made up of dark plastic. The activity was observed at lighted place (360 lux) using a 60 W white light bulb. The compartments were of same size with dimensions of 26 × 26 × 26 cm, between the two compartment a door was present (12 cm × 12 cm). In light compartment the animal was placed and the degree of anxiety was assessed by observing the time spent in the light compartment, cutoff time was 5 min [28].

### Biochemical analysis

Estimation of lipid peroxidation (LPO) was measured as malondialdehyde (MDA) levels and is reported as μmol/g of brain. Brain superoxide dismutase (SOD) activity is reported as U/g of brain. Catalase (CAT) activity was measured as consumption of H_2_O_2_ and is expressed as μmol/min/g of brain. Glutathione peroxidase (GPx) activity is reported as μmol/min/g of brain and reduced glutathione (GSH) levels is reported as μmol/g of brain. These assays were performed as described previously [30]. The amount of fragmented DNA was determined as described previously by Wu et al. [35] with slight modifications [32]. Data are presented as percent fragmented DNA of the total DNA.

### Neurochemical analysis

Acetylcholinesterase (AChE) activity was measured in hippocampus and cortex and is presented as μmol/min/g of tissue. Acetylcholine (ACh) levels were estimated in hippocampus and cortex and are presented as μmol/g of tissue [30]. For determination of biogenic amines in hippocampus and cortex, homogenization of frozen brains was carried out in an extraction medium using an electrical homogenizer (Polytron; Kinematica). The neurochemical analysis was done to assess concentrations of 5-hydroxytryptamine (5-HT), dopamine (DA), and their metabolites 5-hydroxyindoleacetic acid (5-HIAA) and dihydroxyphenyl acetic acid (DOPAC) in brain as described previously [36]. Reversed-phase High Performance Liquid Chromatography (HPLC) with an electrochemical detector (Schimadzu LEC 6 A detector) was performed to detect levels of biogenic amines in brain samples. The EC detector was operated at a potential of +0.8 V. The stationary phase used for separation is a 5-μ Shim-pack ODS column having an internal diameter of 4.0 mm and a length of 150 mm. The mobile phase that passes through a column with a pump pressure of 2000–3000 psi contains OSS (0.023%) in 0.1 M phosphate buffer at pH 2.9.

### Histopathological analysis

Histopathological studies were performed in hippocampus and cortex tissues. Hematoxylin and Eosin (HE) staining following the method of Thenmozhi et al. [8] was used for slide preparation. The histopathological alterations were then viewed and recorder under a light microscope at 400 × magnification.

### Isolation of RNA and analysis of acetylcholinesterase gene expression by RT-PCR

AChE gene expression was determined in hippocampus of rats using reverse transcriptase method. For this purpose, total RNA was isolated from hippocampal region using one step RNA reagent according to the standard method [37]. Integrity of RNA was checked on 1% agarose gel whereas, concentration and purity of RNA was determined using UV–VIS spectrophotometer (Thermo Scientific, USA). Extracted RNA was then treated with DNase to remove any possible traces of genomic DNA. Complementary DNA was synthesized from 1 μg of RNA with the help of RevertAid^™^ First Strand cDNA Synthesis Kit according to the manufacturer’s protocol. cDNA was then used to amplify GAPDH and AChE gene using the specific primer (Table 2) for 35 cycles using the following program; 95°C for 1 min; annealing temperature of respective primer for 45 s; extension at 72°C for 1 min; final extension at 72°C for 10 min. The specific amplicon was synthesized using DreamTaq Green PCR Mastermix. The PCR amplicon was resolved on 1% agarose gel and visualized by ethidium bromide. Bands were then quantified by image J software. The results are expressed as the ratio of AChE amplicon and GAPDH amplicon.

**Table 2:** The sequences of primers used for RT-PCR.

Gene	Primer	Product size	Annealing temperature
AChE	F:CCACCTGAGAGATGCCATGA	225 bp	57°C
	R:ACGGTGTAGTTCAGTGAGGG		
GAPDH	F:ACCACAGTCCATGCCATCAC	452 bp	61°C
	R:TCCACCACCCTGTTGCTGTA		

### Statistics

One-way ANOVA following Tukey’s test for post-hoc analysis was used to compare the data obtained from pre-treatment effects of NAR on AD-like animal model on various behaviors, biochemical and neurochemical estimations. Non-parametric pair wise comparison was used to compare data obtained from nesting behavior. One-way ANOVA repeated measure design with Bonferroni test was used to analyze the data of MWM training sessions.

## Results

### Effect of pre-treatment of NAR on cognitive functions in AD-like animal model

**Fig 2:**
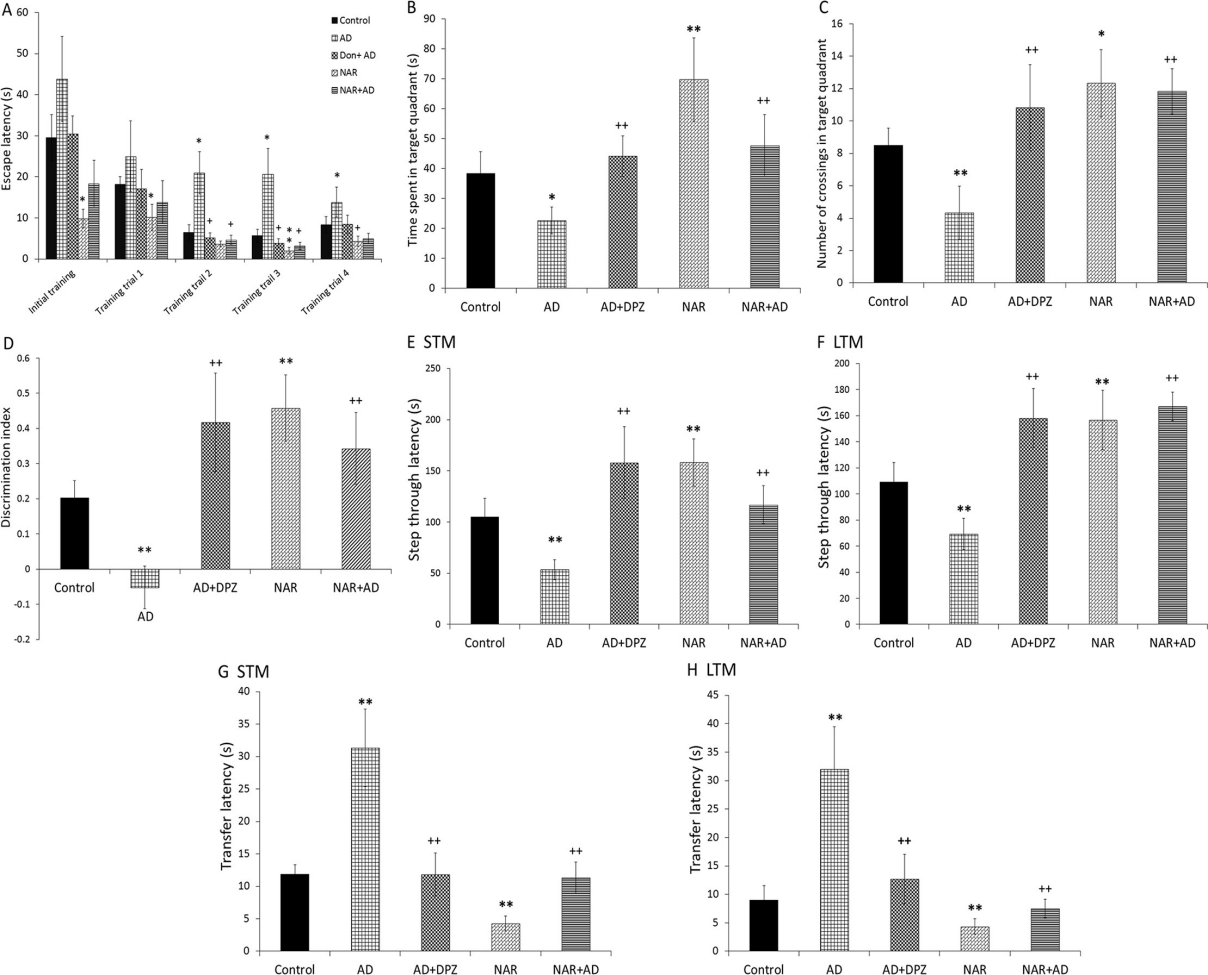
Pre-treatment effects of naringenin prevented cognitive dysfunctions in AD-like animal model. In Morris water maze memory was assessed in terms of (A) acquisition during training sessions and (B) time spent and (C) number of entries in target quadrant during probe trail. (D) Discrimination index was observed in novel object recognition task. Passive avoidance task was used to monitor step through latency during (E) short term (STM) and (F) long term memory (LTM) sessions. In elevated plus maze transfer latency was also monitored during (G) STM and (H) LTM assessment. Data is presented as mean ± SD (n = 6). Data was analyzed by post-hoc analysis following one way ANOVA. Significant differences were obtained *p<0.05, **p<0.01 from control rats; ^++^p<0.01, +p<0.05 from AD-like model rats.

Effect on cognitive functions was analyzed by one-way ANOVA following Tukey’s post-hoc analysis. Significant effect of treatment was found on time spent in target quadrant [F_(4, 25)_ = 20.69, p<0.01] and number of target quadrant crossing [F_(4, 25)_ = 18.98, p<0.01] in MWM (Fig 2B and 2C). AD-like model rats showed a significant decreased (p<0.05) time spent in target quadrant and number of target quadrant crossing (p<0.01) as compared to that of control rats. DPZ treatment in AD-like model rats significantly increased (p<0.01) time spent in target quadrant and number of target quadrant crossing as compared to that of AD rats. NAR treatment significantly increased time spent in target quadrant (p<0.01) and number of target quadrant crossing (p<0.05) as compared to that of controls. NAR pre-treatment in AD-like model rats significantly increased (p<0.01) time spent in target quadrant and number of target quadrant crossing in comparison to that in AD rats. Data for training sessions was also analyzed by one-way repeated measure with Bonferroni post-hoc test. Statistical analysis showed delayed learning ability in AD-like model as evident by increased escape latency (p<0.05) in AD rats as compared to control animals in all training sessions. NAR treatment showed improved acquisition (p<0.05) during training sessions when compared with AD-like model rats (Fig 2A).

ANOVA showed significant effects on recognition memory in terms of discrimination index [F_(4, 25)_ = 28.32, p<0.01] in NOR as shown in Fig 2D. Rats recognition memory was significantly decreased (p<0.01) in AD-like model rats in terms of discrimination index as compared to that in control rats. DPZ treatment in AD-like model rats significantly increased recognition memory compared to that of AD-like model rats (p<0.01). NAR treatment significantly enhanced rats recognition memory (p<0.01) in NOR as compared to that in control rats. NAR pre-treatment in AD-like model rats significantly increased recognition memory in terms of discrimination index as compared to that in AD-like model rats (p<0.01) in NOR.

Associative memory was assessed in PAT. ANOVA showed significant effect of treatment on associative memory of rats in terms of step through latency in STM [F_(4, 25)_ = 22.53, p<0.01] and LTM [F_(4, 25)_ = 36.94, p<0.01] in PAT (Fig 2E and 2F). AD-like model rats showed significantly decreased step through latency in STM (p<0.01) and LTM (p<0.01) as compared to that of control rats. DPZ treatment in AD-like model rats significantly increased step through latency in STM (p<0.01) and LTM (p<0.01) compared that in to AD rats. NAR treatment significantly increased step through latency in STM (p<0.01) and LTM (p<0.01) as compared to that in control rats. NAR pre-treatment in AD-like model rats significantly increased step through latency in STM (p<0.01) and LTM (p<0.01) as compared to AD rats.

EPM was used to assess memory retention. The ANOVA calculations showed significant effects of treatment on memory retention in STM [F_(4, 25)_ = 54.87, p<0.01] and LTM [F_(4, 25)_ = 42.05, p<0.01] as shown in Fig 2G and 2H, respectively. In AD-like model rats significant increase in transfer latency in STM (p<0.01) and LTM (p<0.01) was observed as compared to control rats. DPZ treatment in AD-like model rats significantly decreased transfer latency in STM (p<0.01) and LTM (p<0.01) when compared with AD rats. NAR treatment significantly decreased transfer latency in STM (p<0.01) and LTM (p<0.01) as compared to controls. Moreover, NAR pre-treatment in AD-like model rats significantly decreased transfer latency in STM (p<0.01) and LTM (p<0.01) as compared to AD rats.

### Effect of pre-treatment of NAR on executive functions in AD-like animal model

**Fig 3:**
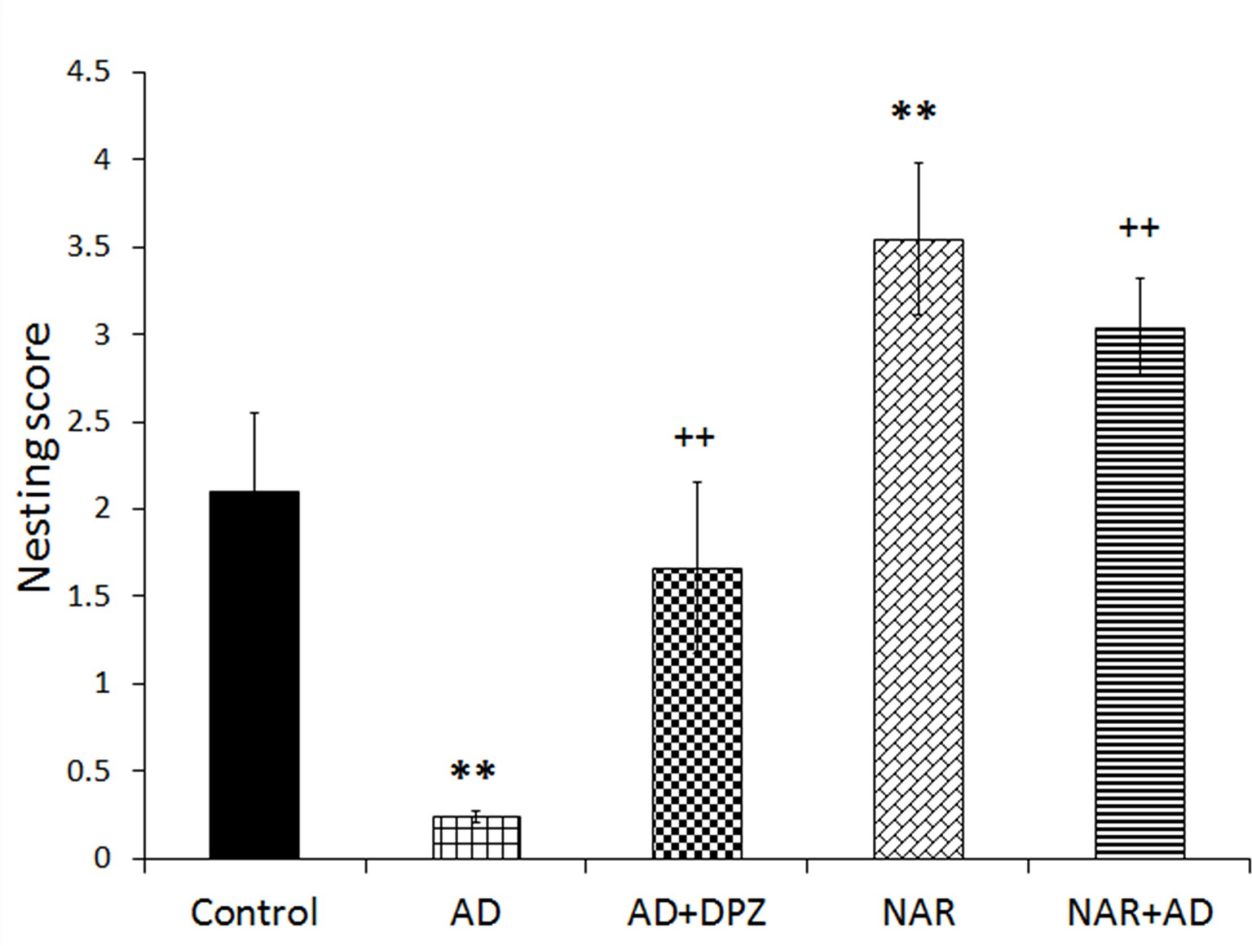
Pre-treatment effect of naringenin improved executive functions in AD-like animal model. Data is presented as mean ± SD (n = 6). Data was analyzed by post-hoc analysis following one way ANOVA. Significant differences were obtained **p<0.01, from control rats; ^++^p<0.01 from AD-like model rats.

Effect of NAR pre-treatment on executive functions of AD-like animal model was analyzed by non-parametric test to compare the effects on nesting behavior. Statistical analysis showed significant effects of treatment on executive functions [F_(4, 25)_ = 69.62, p<0.01]. AD-like model rats showed significant decrease in nesting score (p<0.01) compared to control rats. DPZ treatment in AD-like model rats showed significant increase in nesting score (p<0.01) as compared AD rats. NAR treatment significantly increased nesting score (p<0.01) as compared to controls (p<0.01). NAR pre-treatment in AD-like model rats significantly increased nesting score (p<0.01) in comparison with AD rats as shown in Fig 3.

### Effect of pre-treatment of NAR on psychological functions in AD-like animal

**Fig 4:**
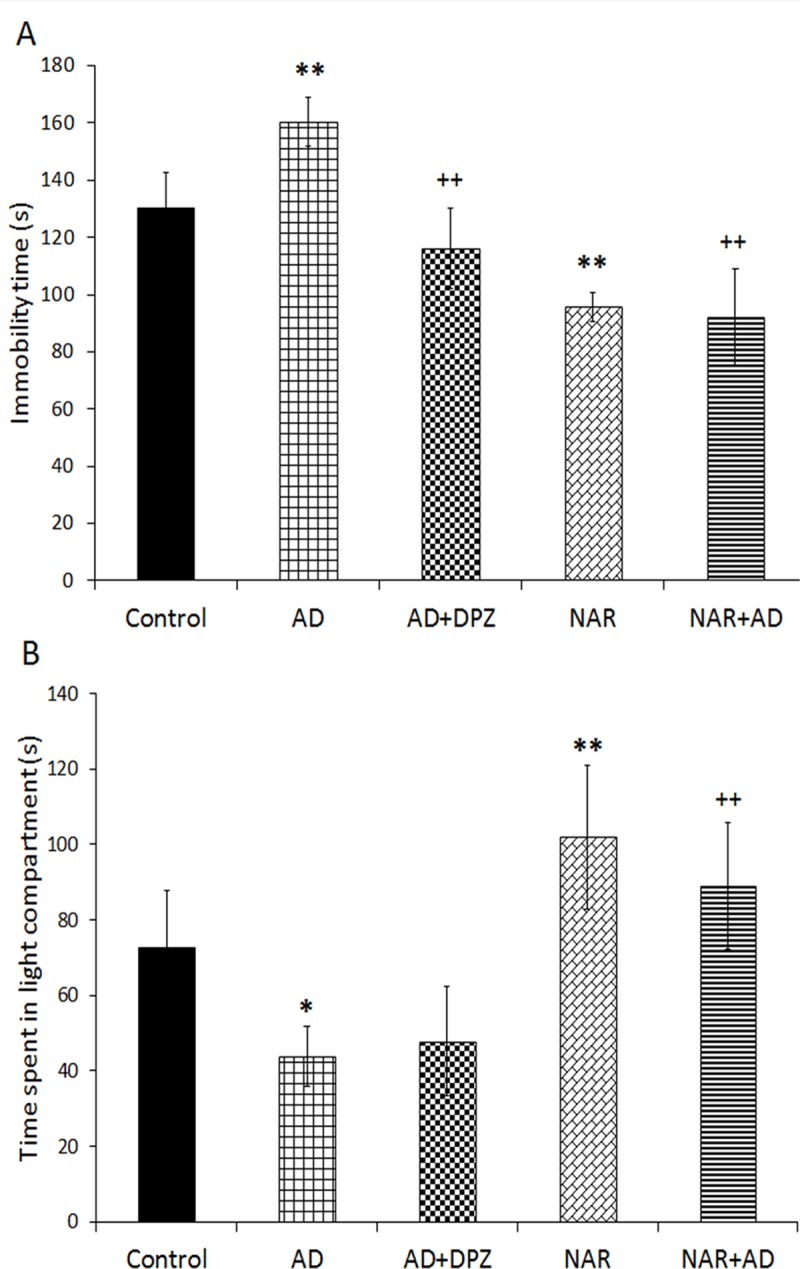
Pre-treatment of naringenin prevented psychological impairment in AD-like animal model. (A) Immobility time in forced swim test. (B) Time spent in light compartment in light/dark transition test. Data is presented as mean ± SD (n = 6). Data was analyzed by post-hoc analysis following one way ANOVA. Significant differences were obtained *p < 0.05, **p<0.01 from control rats; ^++^p<0.01 from AD-like model rats.

Data for psychological functions analyzed by one-way ANOVA showed significant effect of treatment on immobility time [F _(4, 25)_ = 31.82, p<0.01] in FST. AD-like model rats showed significant increase in immobility time (p<0.01) as compared to control rats. DPZ treatment in AD-like model rats significantly (p<0.01) reduced immobility time compared to AD rats. NAR treatment significantly decreased immobility time (p<0.01) as compared to controls. NAR pre-treatment in AD-like model rats significantly decreased immobility time (p<0.01) compared to AD rats as shown in Fig 4A. Significant effect of treatment was found on time spent in light compartment [F_(4, 25)_ = 21.26, p<0.01]. AD-like model rats showed significant decreased time spent in light compartment (p<0.05) as compared to control rats. DPZ treatment in AD-like model rats showed comparable results to AD rats. NAR treatment significantly increased time spent in light compartment (p<0.01) compared to controls. NAR pre-treatment in AD-like model rats significantly increased time spent in light compartment (p<0.01) in comparison with AD rats (Fig 4B).

### Effect of pre-treatment of NAR on brain oxidative stress status in AD-like animal model

**Fig 5:**
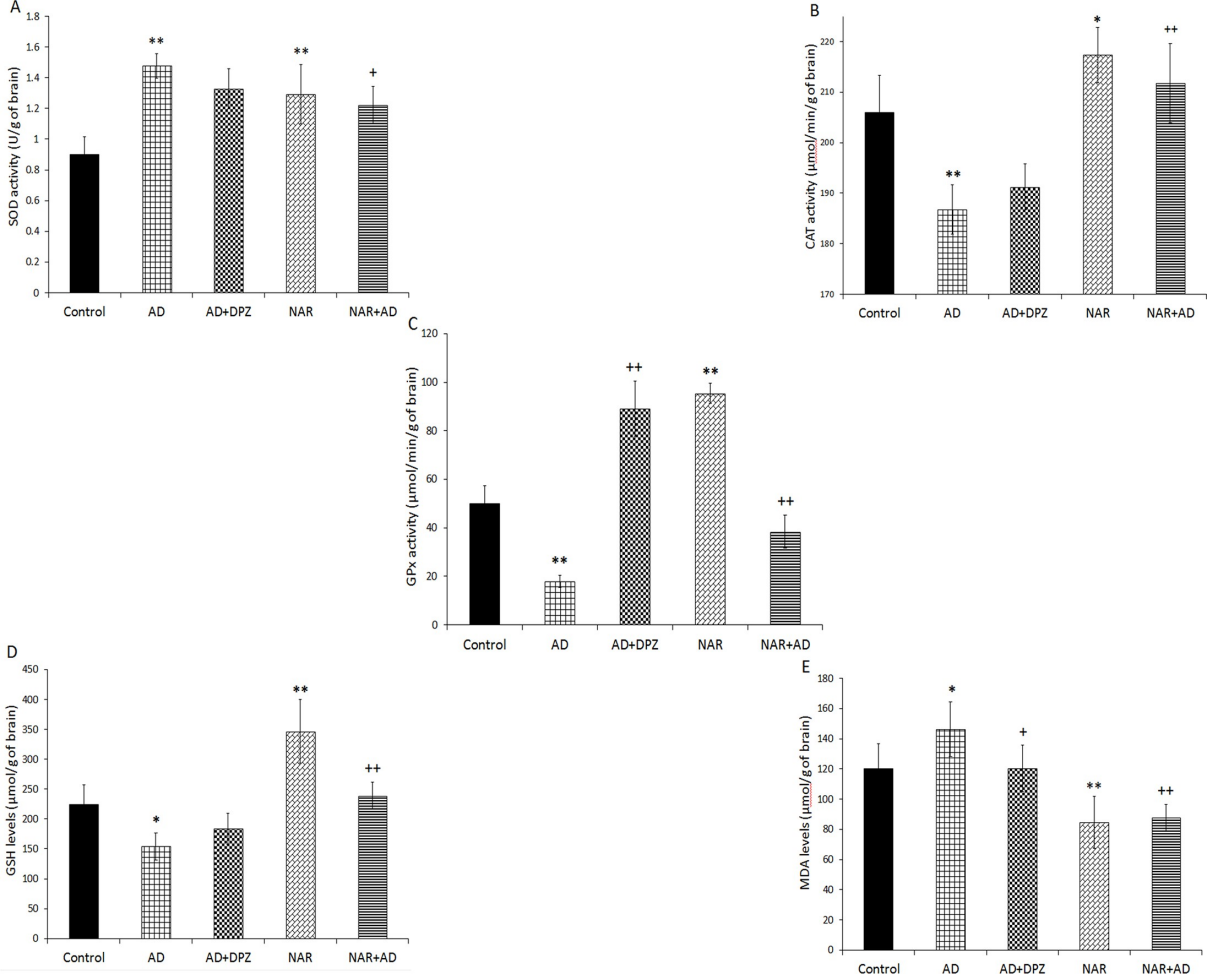
Pretreatment effects of naringenin prevented brain oxidative stress status in AD-like animal model. (A) Superoxide dismutase activity (B) catalase (C) glutathione peroxidase (D) reduced glutathione levels (E) malondialdehyde levels. Data is presented as mean ± SD (n = 6). Data was analyzed by post-hoc analysis following one way ANOVA. Significant differences were obtained *p<0.05, **p<0.01 from control rats; ^+^p<0.05, ^++^p<0.01 from AD-like model rats.

The ANOVA showed significant effects of treatment on brain SOD activity [F_(4, 25)_ = 15.42, p<0.01], CAT activity [F_(4, 25)_ = 27.33, p<0.01], GPx activity [F_(4, 25)_ = 132.10, p<0.01], brain GSH [F_(4, 25)_ = 28.72, p<0.01], and MDA levels [F_(4, 25)_ = 16.27, p<0.01]. SOD activity was also significantly (p<0.01) increased in AD model rats (Fig 5A) as compared to that of controls. NAR treatment significantly enhanced SOD activity (p<0.01) as compared to controls. SOD activity was significantly (p<0.05) decreased in NAR+AD rats when compared with AD group. CAT activity was (p<0.01) significantly decreased in AD model rats than controls. NAR treatment significantly enhanced CAT activity (p<0.05) than control rats. CAT activity was (p<0.01) significantly increased in NAR+AD rats than AD model rats (Fig 5B). GPx activity was significantly (p<0.01) decreased in AD model rats than controls. NAR pre-treatment significantly enhanced GPx activity (p<0.01) compared to controls. GPx activity was significantly increased in AD+DPZ and NAR+AD (p<0.01) rats as compared to AD model rats (Fig 5C). GSH concentration was significantly (p<0.05) decreased in AD model rats compared to control rats. NAR pre-treatment significantly enhanced GSH concentration (p<0.01) compared to controls. GSH concentration was significantly increased in NAR+AD (p<0.01) rats compared to AD model rats (Fig 5D). MDA levels were significantly increased in AD model rats (p<0.05) and decreased in NAR group (p<0.01) as compared to control rats. MDA levels were significantly decreased in AD+DPZ (p<0.05) and NAR+AD (p<0.01) compared to AD-like model rats (Fig 5E).

### Effect of pre-treatment of NAR on % DNA fragmentation in AD-like animal model

**Fig 6:**
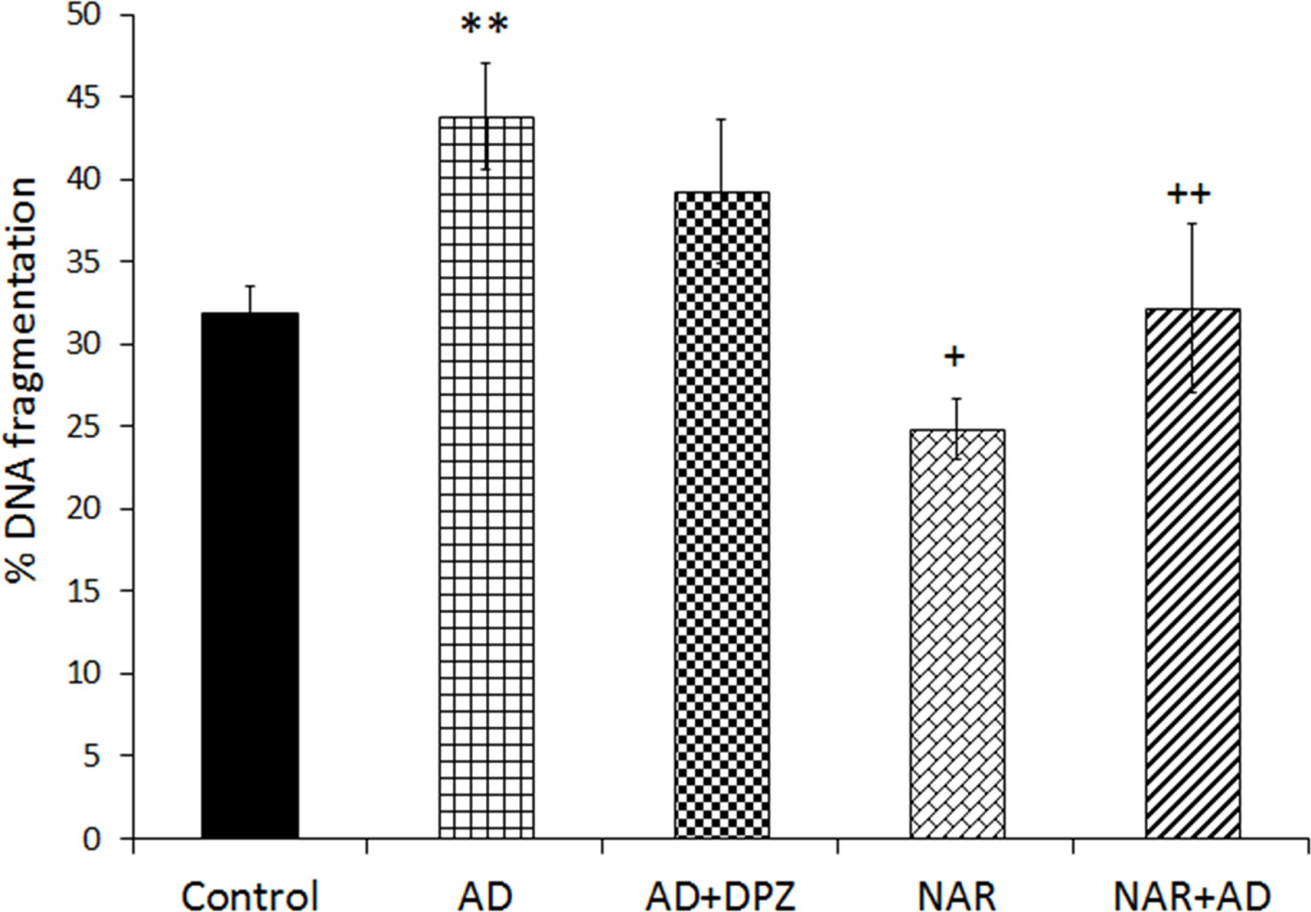
Pretreatment of naringenin prevented % DNA fragmentation in AD-like animal model. Data is presented as mean ± SD (n = 6). Data was analyzed by post-hoc analysis following one way ANOVA. Significant differences were obtained **p<0.01 from control rats; +p<0.05, ^++^p<0.01 from AD-like model rats.

Data showing % DNA fragmentation in AD-like animal model was also analyzed by one-way ANOVA following Tukey’s post-hoc analysis. Current treatment induced significant effect on % DNA fragmentation [F _(4, 25)_ = 26.02, p<0.01]. In the present work, increased % DNA fragmentation compared to control was found in (p<0.01) AD rat model. Decreased % DNA fragmentation (p<0.05) was observed in NAR treated rats compared to controls. NAR treatment significantly protected DNA fragmentation (p<0.01) in NAR+AD rats compared to AD (Fig 6).

### Effect of pre-treatment of NAR on cholinergic functions in AD-like animal model

**Fig 7:**
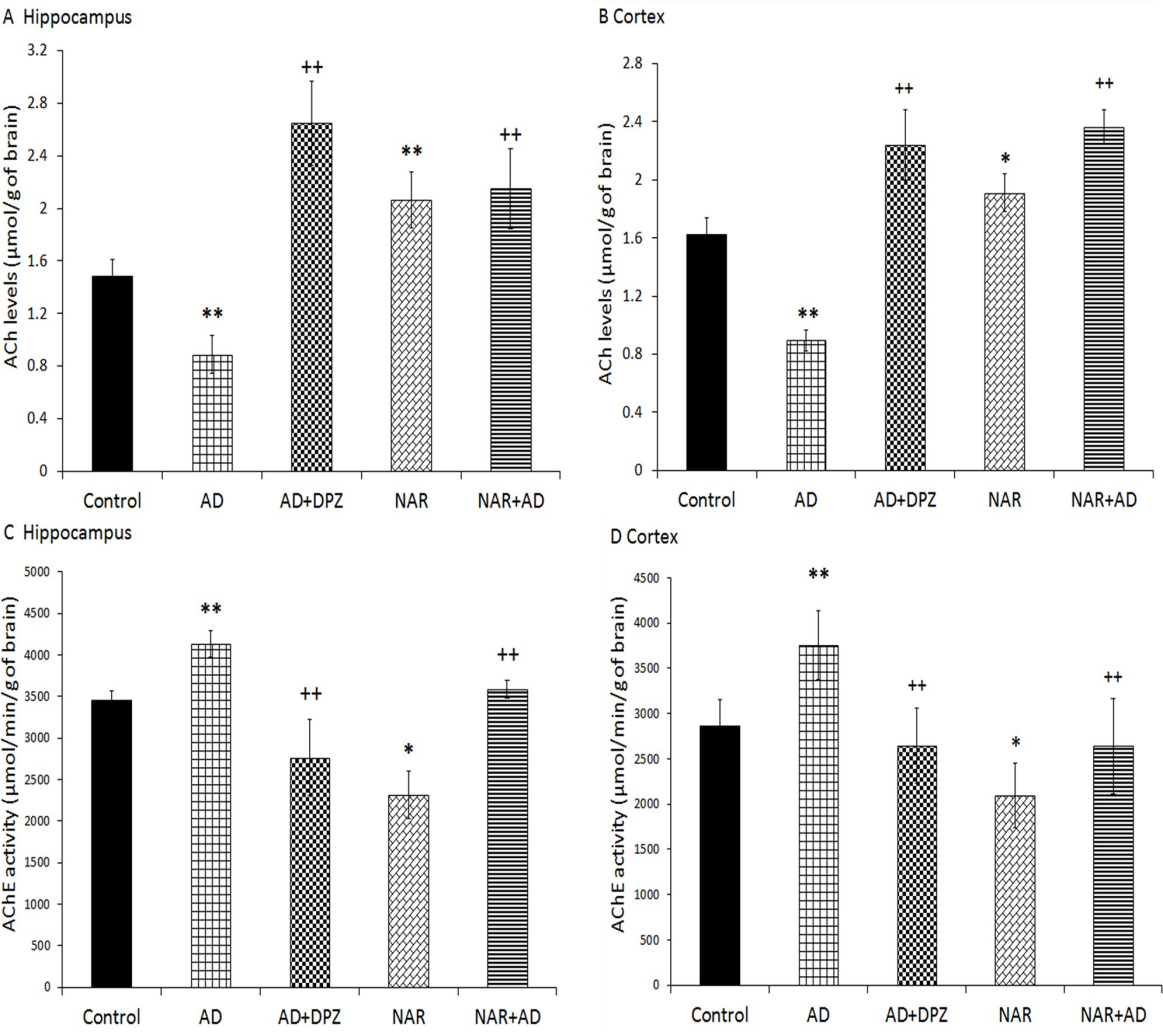
Pretreatment of naringenin prevented cholinergic dysfunctions in AD-like animal model. (A) Acetylcholine levels in hippocampus and (B) in cortex. (C) Acetylcholinesterase activity in hippocampus and (D) in cortex. Data is presented as mean ± SD (n = 6). Data was analyzed by post-hoc analysis following one way ANOVA. Significant differences were obtained *p<0.05, **p<0.01 from control rats; ^++^p<0.01 from AD-like model rats.

The treatment used in the current study produced significant effects on ACh concentration in hippocampus [F_(4, 25)_ = 48.96, p<0.01] and in cortex [F_(4, 25)_ = 93.38, p<0.01]. The treatment also produced significant effect on AChE activity in hippocampus [F_(4, 25)_ = 45.01, p<0.01] and in cortex [F_(4, 25)_ = 13.53, p<0.01]. In the present work, decreased ACh concentration (p<0.01) in hippocampus and cortex was found in AD model rats compared to control. NAR pre-treatment significantly increased ACh concentration (Fig 7A and 7B) in hippocampus (p<0.01) and cortex (p<0.05) as compared to control rats. With comparison to AD model rats, a significant increased (p<0.01) ACh concentration found in AD+DPZ and NAR+AD rats in hippocampus and cortex. In the present work, an increased AChE activity (p<0.01) was found in hippocampus and cortex of AD model rats compared to controls. The NAR treatment significantly decreased (p<0.05) AChE activity in hippocampus (Fig 7C) and cortex (Fig 7D) compared to controls. Compared to AD model rats, a significantly decreased (p<0.01) AChE activity was found in AD+DPZ and NAR+AD rats in hippocampus and cortex.

### Effect of pre-treatment of NAR on hippocampal AChE gene expression in AD-like animal model

**Fig 8:**
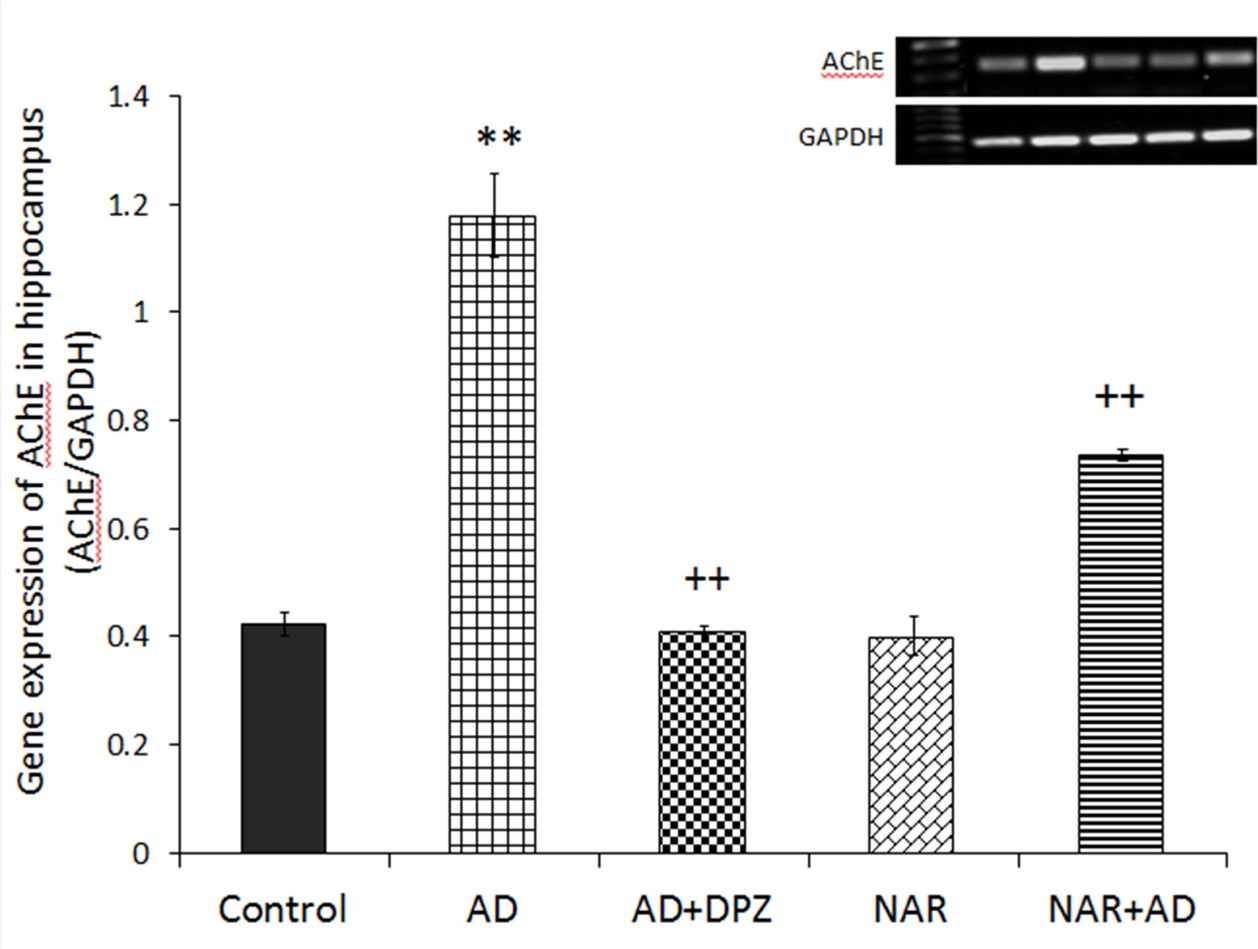
Pretreatment of naringenin prevented increased hippocampal AChE gene expression in AD-like animal model. Data is presented as mean ± SD (n = 3). Data was analyzed by Tukey’s post-hoc analysis following one way ANOVA. Significant differences were obtained **p<0.01 from control rats; ^++^p<0.01 from AD-like model rats.

Statistical data showed significant effect of treatment on AChE gene expression in hippocampus [F_(4,10)_ = 220.38, p<0.01]. Post-hoc analysis by Tukey’s test showed a significant increase in expression of AChE gene (Fig 8) in AD-like model rats as compared to control animals (p<0.01). Whereas, pre-treatment with NAR significantly decreased (p<0.01) expression of AChE gene in NAR+AD group when compared with the rats of AD-like model. AD+DPZ group showed reduced gene expression (p<0.01) as compared to AD rats.

### Effect of pretreatment of NAR on serotonergic functions in AD-like animal model

**Fig 9:**
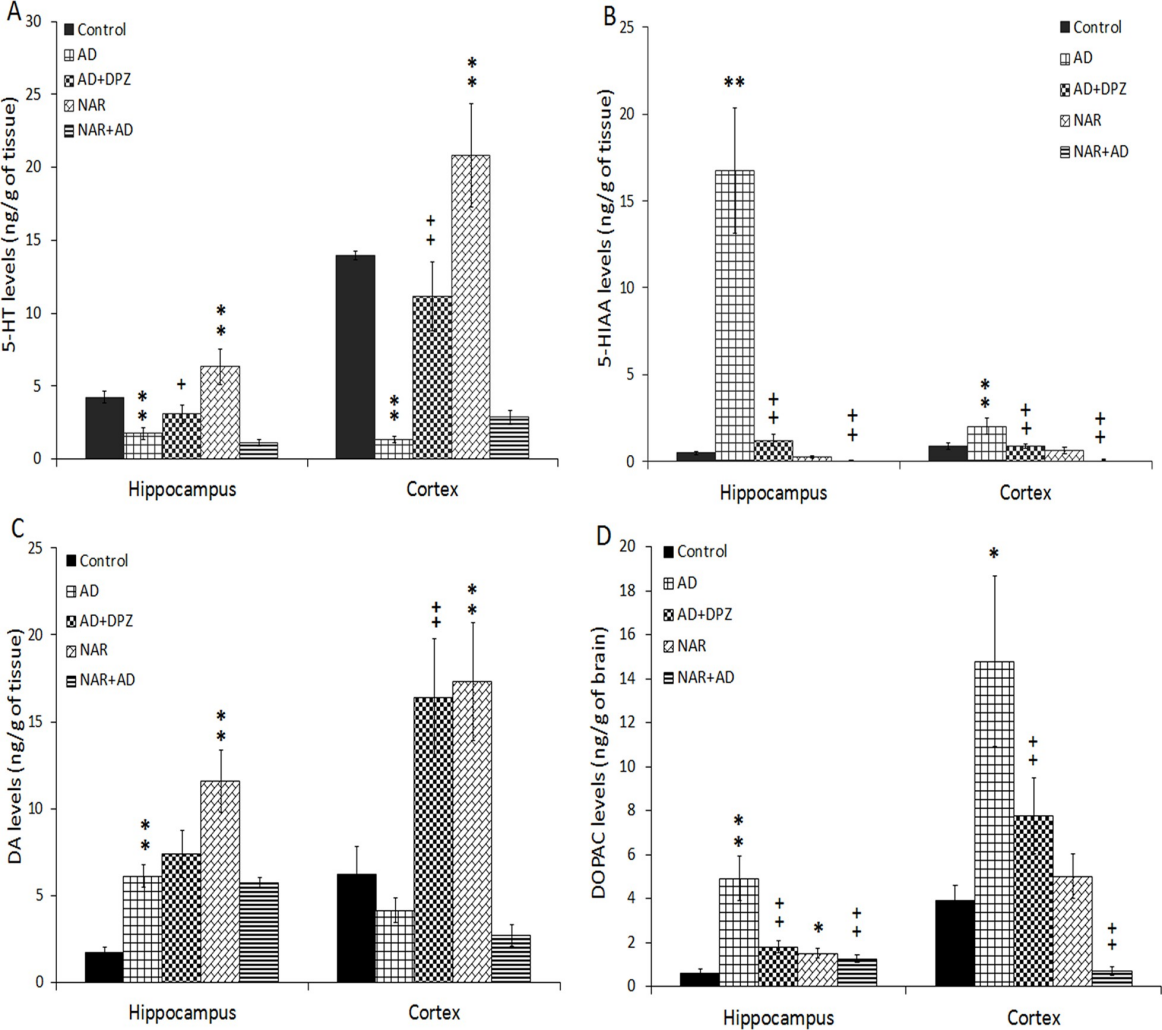
Pretreatment of naringenin prevented decline in serotonergic and dopaminergic functions in AD-like animal model. (A) 5-HT, (B) 5-HIAA, (C) DA and (D) DOPAC levels in hippocampus and cortex were estimated. Data is presented as mean ± SD (n = 6). Data was analyzed by post-hoc analysis following one way ANOVA. Significant differences were obtained *p<0.05, **p<0.01 from control rats; ^+^p<0.05, ^++^p<0.01 from AD-like model rats.

Effect of treatment on 5-HT and 5-HIAA concentration in hippocampus and cortex of AD-like animal model by co-administration of AlCl_3_+_D_-gal, was also analyzed by one-way ANOVA following Tukey’s post-hoc analysis. Significant results of treatment was found on 5-HT levels in hippocampus [F_(4, 25)_ = 58.69, p<0.01] and in cortex [F_(4, 25)_ = 104.78, p<0.01] and its metabolite 5-HIAA levels in hippocampus [F_(4, 25)_ = 122.22, p<0.01] and in cortex [F_(4, 25)_ = 50.19, p<0.01] by statistical analysis. In hippocampus decreased 5-HT levels were observed in AD model rats (p<0.01) compared to control rats. 5-HT concentration was (p<0.01) increased in NAR pre-treated rats compared to control rats (Fig 9A). Whereas, an increased 5-HT concentration (p<0.05) was found in AD+DPZ rats compared to AD model rats. In cortex, a decreased 5-HT levels was found in AD model rats (p<0.01) compared to control rats. 5-HT concentration was (p<0.01) increased in NAR treated rats compared to control rats. An increased 5-HT concentration (p<0.01) was found in AD+DPZ rats compared to AD model rats. In hippocampus, an increased 5-HIAA levels were observed in AD model rats (p<0.01) as compared to control rats while a decreased 5-HIAA concentration was (p<0.01) found in AD+DPZ and NAR+AD rats compared to AD rats. In cortex, an increased (p<0.01) 5-HIAA concentration was found in AD model rats compared to control rats. A decreased 5-HIAA concentration was (p<0.01) found in AD+DPZ and NAR+AD rats as compared to AD-model rats (Fig 9B).

### Effect of pre-treatment of NAR on dopaminergic functions of AD-like animal model

ANOVA showed significant results of treatment on DA levels in hippocampus [F_(4, 25)_ = 67.29, p<0.01] and cortex [F_(4, 25)_ = 43.61, p<0.01] and on its metabolite DOPAC levels in hippocampus [F_(4, 25)_ = 71.74, p<0.01] and cortex [F_(4, 25)_ = 55.17, p<0.01]. In hippocampus DA concentration was (p<0.01) increased in AD and NAR rats as compared control rats. In cortex an increased DA levels were found in NAR and AD+DPZ (p<0.01) compared to controls and AD rats (Fig 9C), respectively. In hippocampus increased DOPAC concentration was found in AD model (p<0.01) and NAR rats (p<0.05) as compared to controls. A decreased DOPAC concentration was (p<0.01) found in NAR+AD and AD+DPZ rats in comparison with AD-model rats. In cortex an increased DOPAC concentration (p<0.05) was found in AD model as compared to controls. A Decreased DOPAC concentration was (p<0.01) found in AD+DPZ and NAR+AD as compared to AD-model rats (Fig 9D).

### Effect of pre-treatment of NAR on histopathological alterations in AD-like animal model

**Fig 10:**
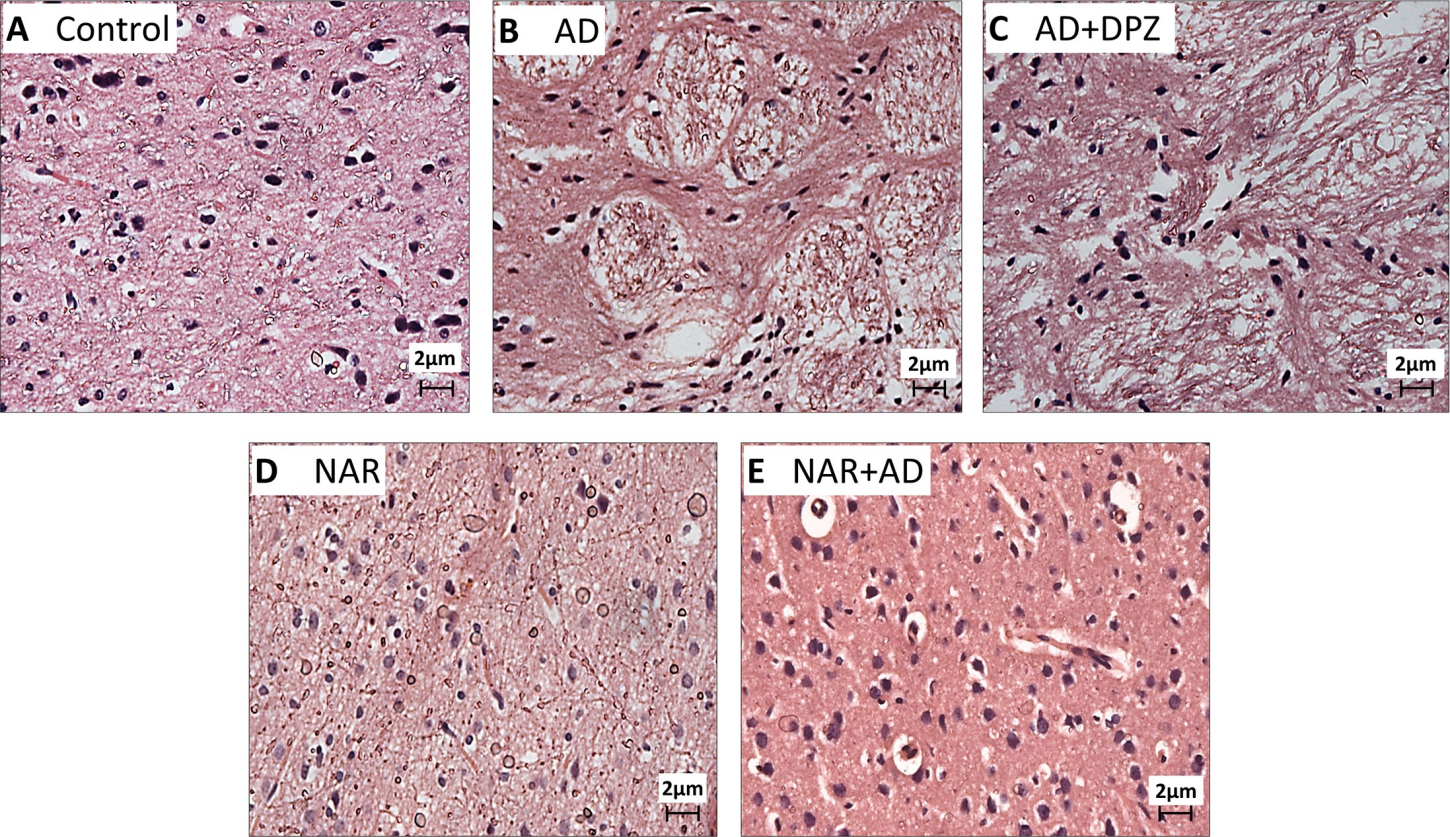
Representative light microphotographs of H & E stained sections from hippocampus of AD-like model and NAR treated rats at 400x magnification. Neurons in AD (B) and AD+DPZ (C) have darkly stained nuclei (long arrows). Neurons have irregular (tangle) and indistinct shape with cytoplasmic vacuolation oedema (arrow head) was also exhibited in sections taken from AD brain samples (B). In NAR pre-treated (D, E) and control group (A) samples: the neurons have intact structure with clear nuclei (long arrows). In NAR+AD (E) neurons have nearly normal morphological appearance with less cytoplasmic vacuolation. In AD (B) extensive neuronal damage and degeneration of neurons and surrounding cells were seen.

**Fig 11:**
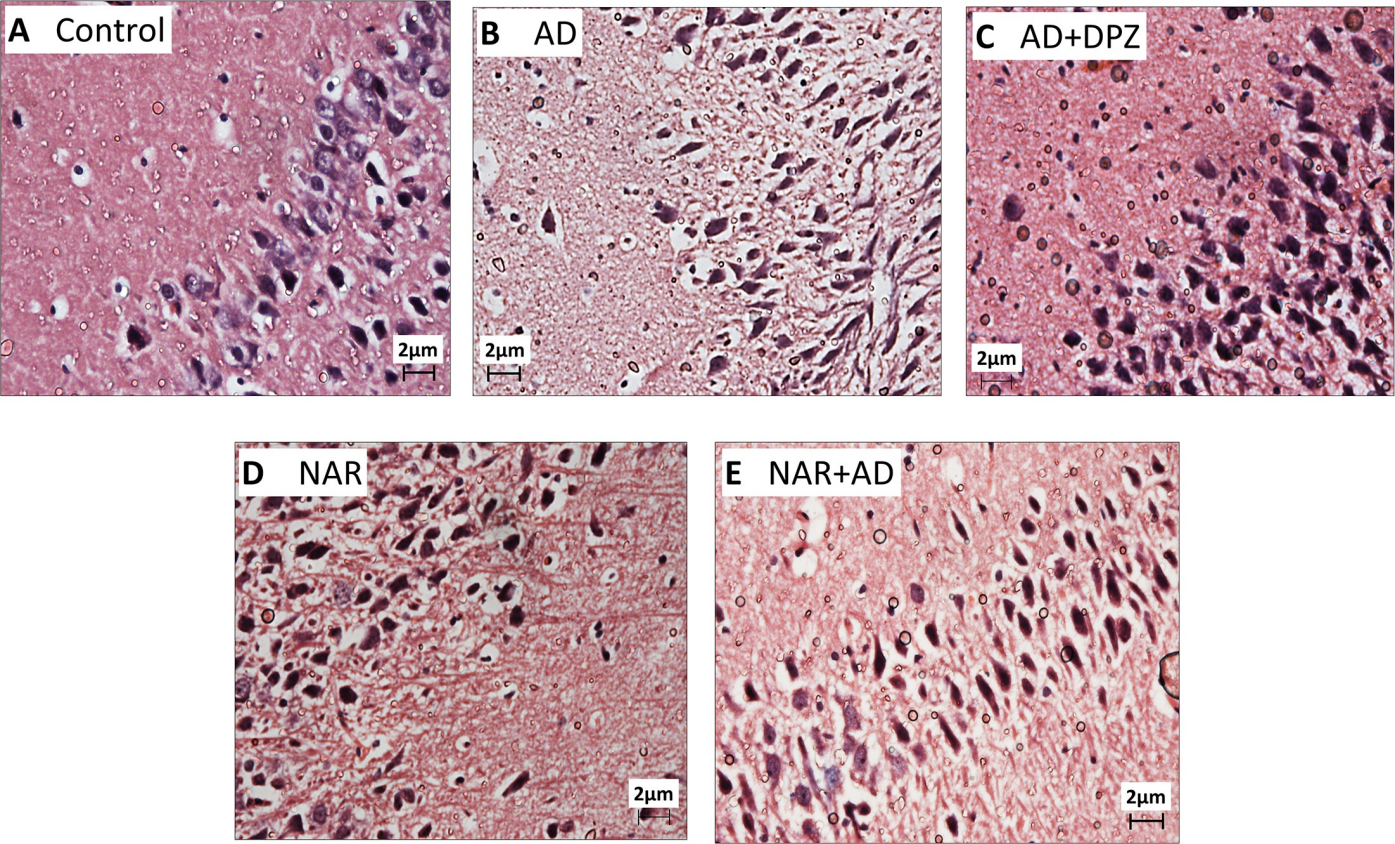
Representative light microphotographs of H & E stained sections from cortex of AD-like model and NAR treated rats at 400x magnification. (A) Control rat brain showing intact circular neurons with clear nuclei (long arrows). Neurons in AD (B) and AD+DPZ (C) showed cytoplasmic vacuolation (long arrows), damaged, irregular and degenerated neurons (arrow head) and perinuclear space. Rats pre-ted with NAR (D, E) had normal neuronal morphology with less cytoplasmic vacuolation and perinuclear space, intact neurons was observed in NAR+AD (E) (long arrows).

Sections from the cortex and hippocampus of AD and AD+DPZ group rats exhibited marked neuronal degeneration. Hippocampal sections of AD and AD+DPZ (Fig 10B and 10C) showed indistinct cell boundaries and irregular damaged cells with cytoplasmic vacuolation. Hippocampal cytoplasm of AD-like model rats was darkly stained and shrunken. Magenta to purple staining is an indication of neurodegeneration. There was also an evidence of perineuronal vacuolation in cortical region of AD and AD+DPZ rats (Fig 11B and 11C). DPZ was not able to prevent the neurodegenerative effects of AlCl_3_+_D_-gal. In hippocampal section of NAR pre-treated rats, nuclear membrane was intact and clear whereas neurons had circular shape and were closely arranged (Fig 10D and 10E). NAR protected neurons against neurodegenerative alternations induced by AlCl_3_+_D_-gal. Cortical sections of rats pre-treated with NAR exhibited normal neuronal morphology (Fig 11D and 11E). Sections from the control rats also showed normal morphology (Figs 10A and 11A).

## Discussion

The AD is characterized with slow and insidious progression of cognitive deterioration and mood disturbance with struggle in daily life activities and decreased social interactions [38]. The primary cause of AD is still unclear, but brain cholinergic dysfunction, oxidative stress, and decreased metabolic rate of neurons are the principle and the most reported pathogenic factors [39,40]. The Oxidative stress mediated neurotoxicity is a main pathological factor in the underlying neurodegenerative mechanism of AD [41]. It is considered as an early event in the occurrence of neurodegenerative disorder and has an imperative part in Aβ-induced cell death in brain [42]. Polyphenols are considered as protective agents against oxidative stress induced neurodegeneration due to their strong antioxidant properties [43]. In our previous work neuroprotective effects of three different polyphenolic compounds NAR, quercetin and curcumin were investigated where NAR acted as the most potent antioxidant among the three tested polyphenols [30]. Hence, the objective of the present study was to evaluate the protective effects of NAR in AD-like animal model induced by co-administration of AlCl_3_ and D-gal.

Rats were pre-treated with NAR, and then exposed to combined treatment of AlCl_3_ and _D_-gal and various behavioral procedures were used to proof NAR as a potent agent to reduce AD-like symptoms in an animal model. DPZ, a selective mixed competitive and non-competitive reversible inhibitor of AChE, is an approved drug to treat mild to moderate symptoms associated with AD by the Food and Drug Administration (FDA) [38]. It was used as a standard therapeutic drug for AD model (positive control) in the current investigation to further strengthen the present hypothesis. AD is the most common cause of dementia in the elderly people and is clinically characterized by cognitive dysfunction, temporal and spatial disorientation, loss of lexical access, and impairment of judgment [44]. Neurodegenerative process in the early stage of AD is reflected well in the form of behavioral abnormalities that may be accompanied by a decreased activity in cortex, in both medial and temporal regions [45]. Rats pre-treated with NAR showed improved spatial memory as indicated by increased time spent and number of crossings in target quadrant during assessment in MWM. Learning ability of AD rats pre-treated with NAR was also improved in MWM. Cognitive abilities of AD model rats were severely affected, consistent with our previous work [29] and also with previous reported studies [5]. Xiao et al. have reported similar behavioral alterations in AD model induced by co-administration of AlCl_3_ and D-gal. Cognitive deficits, decreased cholinergic activity, and AD-like lesions in brain, were also observed after combined administration of AlCl_3_ and D-gal [46]. Consistent with previous studies, treatment with DPZ improved cognitive functions in AD-like model rats [47,48]. In the present investigation NOR was used for assessing recognition memory of rats. It has been reported that NOR can be used to determine the damaging effects of different pharmacological agents on brain by measuring preference of novelty in rats [49]. Results of the study showed that NAR pre-treatment improved recognition memory in rats. The AD patients are not able to maintain perceptual memories, and their visual recognition memory is also severely affected due to excessive neurodegeneration especially in cortical regions [50]. We found that recognition memory and learning capacities were also severely affected in AD model rats similar with those observed in AD patient. However, NAR pre-treatment significantly protected rat recognition memory possibly by protecting cortical neurodegeneration produced by AlCl_3_ and D-gal. Regions that are mainly affected in AD brain are cortex, hippocampus, and amygdala [51,52]. We found that in AD-like model rat’s associative memory was severely impaired whereas NAR protected rat’s associative memory in AlCl_3_+D-gal induced cognitive deficits in PAT. Marked increase in transfer latency was observed in AD-like model rats in EPM while NAR pre-treatment significantly enhanced working memory in NAR+AD rats as indicated by decreased transfer latency compared to that of AD-like model rats. Similar results were reported following Aβ vaccination in transgenic mouse model of AD [53]. A study has shown that Aβ peptide vaccination significantly protected and enhanced mice working memory in radial arm water maze [53]. NAR pre-treatment also improved working memory in AD-like model rats. Results obtained from MWM, NOR, PAT and EPM analysis, therefore, strongly suggest cognitive enhancing effects of NAR. Previously NAR has shown the ability to reduce Aβ levels, prevent hippocampal apoptosis and improve spatial working memory in Aβ injected rats [14].

It has been reported that along with learning deficits, neuropsychiatric symptoms are important in primary care giving of AD patients [54]. Nest building paradigm was used to monitor non-learned behavior in rats. Nest building is characterized as a specie specific test and it is a form of active interaction with surrounding for temperature control and shelter. Nest building is normally used to evaluate executive functions [34]. Compared to rats in other groups, AD-like model rats built poor quality nest whereas NAR pre-treated rats built near to perfect nest. Nesting behavior was evaluated in terms of nesting score and highest nesting score was observed in NAR pre-treated rats. Nest building behavior is based on step by step organization and planning and it is sensitive to prefrontal cortex lesions [34]. A possible association may be drawn here between the nesting behavior and loss of initiative observed in AD patients.

Psychological functions that were monitored include anxiety and depression. Different life stressors such as financial difficulties and personal loss are important contributing factors for depression [55]. Depression in various neurological disorders like AD is also well reported and it is linked with negative consequences in AD and patients caregivers [56]. AD-like model rats showed depressogenic behavior in terms of increased immobility time in FST in the present investigation. This depression-like behavior was not observed in NAR pre-treated rats. Anxiogenic behavior was clearly observed in AD-like model rats whereas NAR pre-treatment produced anxiolytic effects. The AD rats on NAR pre-treatment spent more time in light compartment compared to that of untreated AD-like model rats. DPZ treatment was not able to ameliorate anxiogenic effects and hence psychological alterations in AD-like model rats. Based on present results it is suggested that NAR may be considered as a suitable drug to not only improve cognitive but also psychological dysfunctions and related symptoms linked with AD.

Increased free radical generation is linked to disruption of mitochondrial respiratory chain that results in excessive generation of free radicals and ultimately leads to cellular damage [57]. SOD generates OH^-^ radicals that are scavenged and eliminated by the GPx and CAT [58]. NAR pre-treatment significantly enhanced antioxidant enzyme activities by inhibiting LPO and by free radicals scavenging properties. Enzymatic antioxidant activities and non-enzymatic antioxidant concentration was determined in rest of the brain. We found that CAT activity was significantly decreased in AD-like model rats and NAR pre-treatment increased brain CAT activity. Similarly, GPx activity and GSH concentration were markedly decreased in AD-like model rats which is consistent with our previous work [29]. Whereas, increased brain GPx activity and GSH concentration was found in NAR pre-treated rats. Under normal physiological conditions free radicals are normally produced in a cell but they are continuously and efficiently eliminated from the cell by the catalytic action of antioxidant enzymes to keep a homeostatic balance. Any disturbance in the homeostatic balance may lead to deleterious effects on various organs including brain. In the brain they can initiate neurodegenerative process and stimulate natural aging process [59]. Following consequences of oxidative stress, SOD provides first line of defense followed by the activities of CAT and GPx [58]_._ The antioxidant activity of SOD is only useful when it is followed by the antioxidant activities of GPx and CAT [60]. Increased SOD with decreased CAT and GPx activities may produce damaging effects on cell [61,62]. We observed an increased SOD activity in AD-like model rats but this increased SOD activity was not followed by the increased activities of CAT and GPx. As a result, increased LPO levels (increased MDA) was observed in AD-like model rats. However, in NAR pre-treatment increased SOD activity along with increased CAT and GPx activities was observed. Previously, it is shown that flavonoids have regulatory effects on ɤ-glutamyl cysteine synthetase enzyme, which is the main enzyme in the GSH synthesis. It is possible that NAR may have stimulated ɤ-glutamyl cysteine synthetase expression to increase brain GSH concentration. Further experiments are required to investigate the direct effect of NAR on ɤ-glutamyl cysteine synthetase.

Undergoing the normal aging process, brain undergoes various functional and structure modifications that affect dendritic and synaptic connections, blood circulation, and metabolism of different neurotransmitters that result in impaired neurotransmission [44]. Such alterations are reflected in various behaviors including sleep, learning and memory, and motor and sensory systems. Along with this, during aging cholinergic and catecholaminergic systems are also severely affected [44]. Disturbance in cholinergic neurotransmission strongly correlates with the extent of dementia and severity of neuropathological changes associated with AD [63]. Impaired cholinergic functions in brain are considered to be involved in cognitive dysfunction associated with aging [39]. Role of cholinergic function in cognition was first recognized after cholinergic antagonists were found to impair learning and memory abilities [64]. The cholinergic functions in terms of concentration of ACh and AChE activity was estimated in cortex and hippocampus. ACh levels were markedly declined in hippocampus as well as in cortex in AD-like model rats. However, NAR pre-treatment significantly increased ACh levels in hippocampus and cortex. In our previous work, we found improved cholinergic functions in term of increased ACh levels and decreased AChE activity in brain after 24 hours of NAR administration [30]. Umukoro et al. found attenuating effects of NAR against behavioral derangements by inhibition of AChE activity [65]. In the present study NAR improved cognitive functions in rats mainly by inhibition of AChE activity in brain as already reported [25]. Treatment of DPZ to AD-like model rats also showed significantly increased ACh levels in cortex and hippocampus. AChE activity was markedly decreased in NAR pre-treated rats. A significant increase in expression of AChE gene and increased AChE activity was observed in AD-like model rats than control rats. Previously it has also been reported that NAR has inhibitory effects on AChE activity and therefore it has the potential to be effective in dementia and associated disorders [25]. Treatment of NAR to AD-like model rats also decreased AChE activity in hippocampus as well as in cortex. Pre-treatment with NAR also significantly decreased expression of AChE gene when compared with the rats of AD-like model. DPZ treatment reduced the activity of AChE in cortex and hippocampus. AD+DPZ also showed reduced gene expression of AChE as compared to AD rats.

Previous work has suggested a higher risk of DNA fragmentation in AD compared to age-matched controls and suggested DNA fragmentation as an indicator of genotoxicity. Nerve cell degeneration is one of the hallmarks of AD and along with neurons, DNA fragmentation also indicates death of oligodentrocytes and microglia in AD brain [66]. Lassman et al. further reported that DNA fragmentation in brain cells of AD was 30% more as compared to age-matched controls [66]. In AD brain large number of nerve cells displayed DNA fragmentation than controls [67]. Similar results were obtained in our present work, % DNA fragmentation was significantly high in the brain of AD-like model rats as compared to control rats. Stadelmann et al. [67] found higher DNA fragmentation in hippocampus of AD brain and they suggested a metabolic disturbance as a key factor in DNA fragmentation and associated genotoxicity in AD brain. Oxidative stress is reported as a pathological factor in DNA fragmentation [68], nuclear and mitochondrial DNA oxidative damage potentially contribute to neurodegenerative mechanism in AD. Increased oxidative stress in terms of increased LPO was observed in AD-like model rats and this may be considered as a possible pathogenic factor in DNA fragmentation observed in brain of AD-like model rats. Various antioxidants have shown protective effects against hepatic and brain LPO and DNA fragmentation [69]. Previously, NAR has shown DNA repairing effects in prostate cancer cells [70] and prevention against DNA damage in alloxan-induced diabetic mice [71]. We also found that NAR pre-treatment significantly protected brain cells from genotoxicity as indicated by a decrease in % DNA fragmentation in brain of AD-like rats after NAR treatment. Protective effects of NAR against DNA fragmentation may be credited to its potent antioxidant properties.

Neurotransmission is a highly dynamic process supported by continuous cycling of neurotransmitters and evaluation of neurotransmitter concentration in brain may provide a mirror image of the status of neurotransmission. Under normal physiological conditions, synthesizing and degrading enzymes of neurotransmitters are not fully activated. Any error in metabolic pathways may result in alteration of neurotransmitter homeostasis [72]. Serotonergic dysfunctions are most prominent in the initial stages of AD and reduced activity of 5-HT and its metabolite has been observed in post-mortem AD brain. In temporal cortex of AD patients, depleted 5-HT reuptake sites [73] and depleted 5-HT and its metabolite levels have been observed [74]. Disturbance in both post synaptic and pre synaptic 5-HT system has been observed in AD [73]. Selective depletion in cholinergic neurotransmission results in memory impairment and other cognitive dysfunction while additional serotonergic blockade or disturbance results in the appearance of several behavioural disorders. Combined administration of AlCl_3_+_D_-gal impaired multiple neurotransmitter system i.e. cholinergic, serotonergic and dopaminergic system. The serotonergic functions as 5-HT and 5-HIAA levels and dopaminergic functions as DA and DOPAC levels were measured in hippocampus and cortex. Significantly decreased 5-HT levels were found in hippocampus and cortex of AD-like model rats while NAR pre-treatment significantly increased 5-HT levels in hippocampus and cortex. Treatment of DPZ to AD-like model rats also showed increased 5-HT levels in hippocampus and cortex. However, 5-HIAA levels were found to be increased in AD-like model rats but decreased in cortex and hippocampus of NAR pre-treated rats. Preclinical and clinical studies have also described the role of 5-HT and its receptors in different aspects of learning and memory functions [75,76]. Serotonergic and dopaminergic cells are also highly affected in AD along with cholinergic dysfunctions. Significant decrease in DA and 5-HT levels have been reported following aluminum nitrate exposure at a dose of 25 mg/kg orally once daily for 6 weeks. Aluminum (Al) decreases DA and its metabolite concentration in the brain by altering the activities of DA synthesizing enzymes [77]. Several studies have investigated an association among neurotransmitter levels, Al-induced neurotoxicity and AD [78]. Dopaminergic neurotransmission is also severely affected in AD, as a recent study demonstrated a pathogenic role of dopaminergic dysfunction in cognitive decline in AD [79]. Neurons forming the nigrostriatal pathways undergo various pathologic changes such as formation of neurofibrillary tangles and Aβ plaques, neuronal loss and neuropil threads and also a decrease in concentration of DA. All such alterations have clearly indicated that DA is involved in the pathophysiology of cognitive impairment as well as in non-cognitive symptoms of AD [80]. Consistent with the previous report, we also found that DA levels were significantly decreased in AD-like model rats, while increased DA levels were found in hippocampus and cortex of NAR pre-treated rats. DOPAC levels were significantly increased in AD-like model rats and decreased in hippocampus and cortex of NAR pre-treated and DPZ groups compared to that of control rats. Activities of rate limiting enzymes of DA and 5-HT are reported to be decreased during aging [81]. Previous studies have reported a strong link between oxidative modifications during aging and limited or low activities of synthesizing enzymes of 5-HT and DA. [81]. Such oxidation modifications lead to reduced dopaminergic and serotonergic functions in brain and eventually result in age-associated psychological disorders such as depression, anxiety and stress. During aging oxidative stress initiate apoptosis in nerve cells that lead to cell death and loss of neurons [82]. In present work AD-like model rats injected with combination of AlCl_3_ and D-gal showed increased oxidative stress, which may be the possible reason behind reduced dopaminergic and serotonergic functions. Serotonergic and dopaminergic dysfunctions are due to increase in oxidative stress that initiates apoptotic mechanism in brain and results in dopaminergic and serotonergic neuronal damage as reported earlier [83], that eventually resulting in reduced neurotransmitter levels. Al has strong neurotoxicological potential and its concentration in human brain increases as the age progress. Previous studies support the fact that Al can cross blood brain barrier and accumulates in various brain regions. Cholinergic neurons are particularly more prone to Al associated neurodegeneration as compared to other neurotransmitter system [72] and changes in neuronal morphology is the most reported feature of Al intoxication [84]. Deleterious effects of Al includes structural and functional modification of cytoskeletal proteins that results in accumulation of hyperphosphorylated τ proteins that further leads to formation of neurofibrillary tangles, axonal and dentritic system breakage and associated neurodegeneration. Al exposure initiates apoptotic cell death by inducing damage to mitochondria and endoplasmic reticulum, impairment in energy metabolism, protein phosphorylation imbalance and neuronal excitotoxicity have also been reported following Al exposure [72]. All these deleterious pathways are similar with those generally accepted mechanisms associated with AD. However, the most accepted hypothesis is aggravation of oxidative stress by reducing antioxidant enzyme activities, increasing superoxide oxidation, interfering with transport and storage of iron and exacerbating LPO. Above mentioned neurotoxic mechanism of Al exposure would certainly impair neurotransmission and related processes [72]. To evaluate morphological alterations, in rat's cortex and hippocampus histopathological studies were done. NAR pre-treatment significantly prevented AlCl_3_ and _D_-gal induced histopathological alterations. Findings were further strengthened by reported histopathological results in which the cells were darkly stained in AD-like model rats which clearly indicates oxidative stress [85]. Perinuclear space as well as vacuolization around the neuronal cells was found in cortex and hippocampus of AD model rats. Similar histopathological changes such as diffused gliosis and pericellular edema in cerebral cortex and high level of pyramidal cell degeneration with marked cell distortion was observed in hippocampal slides of Al treated rats [86]. Coban et al. observed marked vacuolar changes, slight edema, and mild inflammatory infiltration in cortical areas of D-gal treated rats and indicate such histopathological changes are due to neurodegenerative process in rat brain [87]. Moreover, disorganized nerve fibres with irregular neurons in the hippocampal CA1 region was also observed following D-gal administration [88]. Kenawy et al. also reported irregular morphology in cortical and hippocampal slides of D-gal rats similar to our observations [89]. Flame-like elongated and degenerated neurons were also observed in hippocampus of AD rats. Khan et al. observed similar histopathological changes in CA1 hippocampal area of intracerebroventricular-streptozotocin (ICV-STZ) induced AD-type model rat. In hippocampal slides of (ICV-STZ) induced AD-type model rat, the neuronal cells were found to be shrunken and dense chromatic nuclei was observed [58]. Hippocampal and cortical cytoplasm of AD-like model rats was darkly stained and shrunken. Magenta to purple staining is an indication of neurodegeneration. In NAR pre-treated rats nuclear membrane was intact and clear whereas neurons had circular shape and were closely arranged. In AD model rats, nuclear membrane was not clear and neurons had flame-like irregular shape. In AD+DPZ rats, damaged and degenerated neurons were observed having irregular and indistinct morphology. One of the main findings of present work is that DPZ was not able to prevent the neurodegenerative mechanism induced by AlCl_3_+_D_-gal. However, NAR not only prevented the behavioral and neurochemical activities but also histopathological alterations and DNA fragmentation in brain of AD-like model rats.

## Conclusion

**Fig 12:**
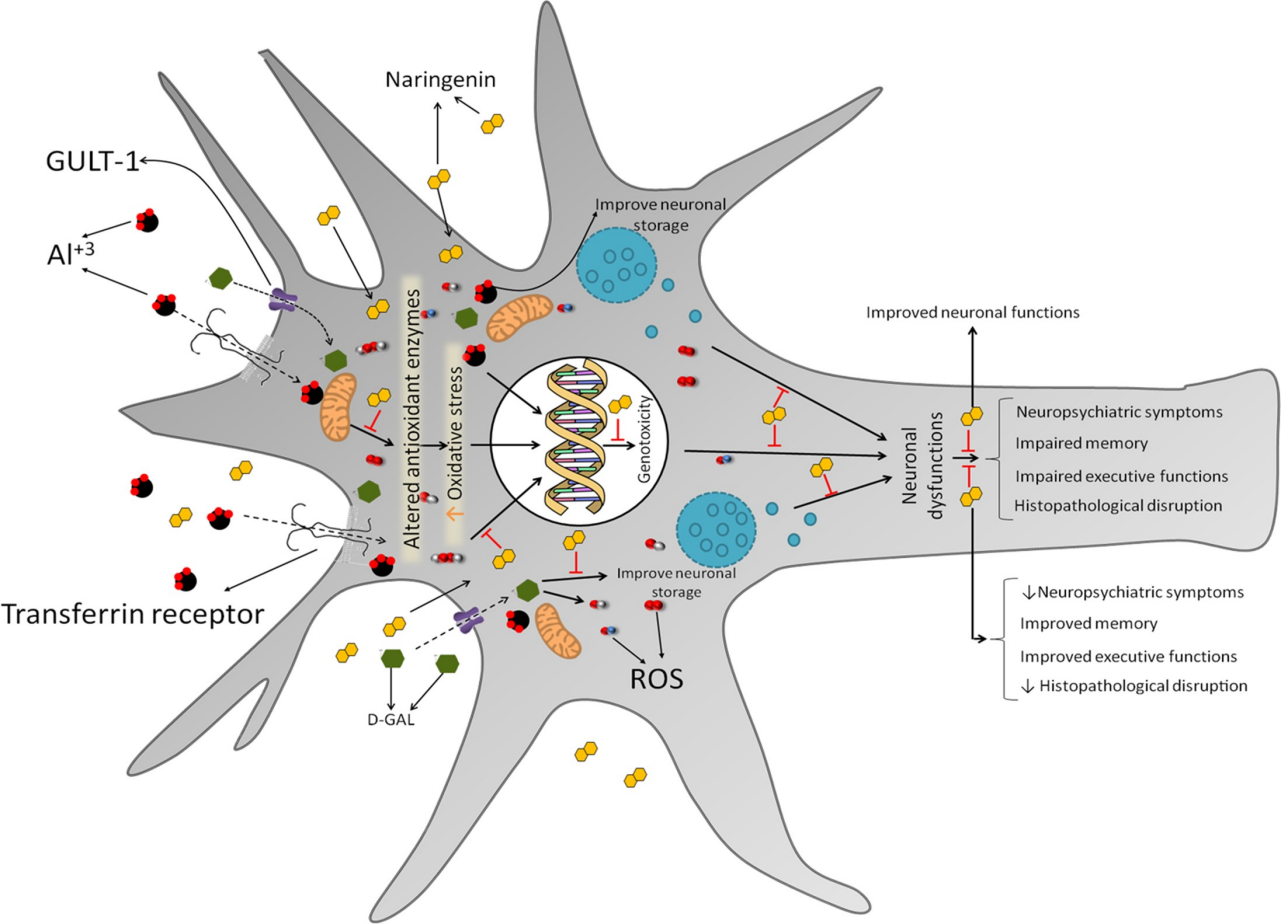
Schematic representation showing the possible mechanism by which naringenin protects brain against AD-like symptoms induced by co-administration of Al and _D_-galactose. The findings of the present study demonstrate the antioxidant potential of NAR evident by reduced oxidative and improved activities of antioxidant enzyme activities following the administration of Al and _D_-galactose. This results in reduced genotoxicity and improved neuronal activity including cholinergic, serotonergic and dopaminergic neurotransmission. Pre-treatment with NAR significantly protects neurobehavioral alterations and improves cognitive, executive and psychological functions in rats. Hence, along with cognitive improvement NAR may be considered as a useful compound in the therapeutic management of behavioral disturbances associated with AD.

The present study suggests that NAR, a potent antioxidant, has an ability to prevent oxidative damage by increasing neuronal antioxidant enzyme activities, preventing LPO and stimulating cholinergic, serotonergic, and dopaminergic neurotransmission (Fig 12). Neuroprotective agents such as NAR could be used in the earlier stage of the disease to stop the progression of the neurodegenerative process. NAR may be considered as an important precursor molecule in the quest to develop novel neuroprotective drugs in therapeutic management of AD. Taken together, it is concluded that NAR pre-treatment significantly protected neurobehavioral alterations and improved cognitive, executive, and psychological functions in rats. Hence, along with cognitive improvement, NAR may be considered as a useful compound in the therapeutic management of behavioral disturbances associated with AD.

## Supporting information

S1 Table Morris water maze test for screening of rats. Values are mean ± SD (n = 6). Non significant difference was obtained following one-way ANOVA.
(DOCX)
