# Supplementary material for: Naringenin protects AlCl3/D-galactose induced neurotoxicity in rat model of AD via attenuation of acetylcholinesterase levels and inhibition of oxidative stress
Source: PLoS One. 2020 Jan 16;15(1):e0227631. doi: 10.1371/journal.pone.0227631 (PMC6964982; doi:10.1371/journal.pone.0227631)
Supplement: S1 Table — Values are mean ± SD (n = 6). Non significant difference was obtained following one-way ANOVA. (DOCX) [file pone.0227631.s001.docx]

**Supporting data**

**Table 1. Morris water maze test for screening of rats.**

| **Groups (n=6)** | **Control** | **AD** | **AD+DPZ** | **NAR** | **NAR+AD** | **Control** |
| --- | --- | --- | --- | --- | --- | --- |
| Time Spent in Target Quadrant | 37.5±10.5s | 41.3±4.6s | 42.3±10.2s | 35.1±11.8s | 39.5±5.8s | 37.3±11.0s |
| Number of Entries in Target Quadrant | 5.8±2.4 | 8.1±2.3 | 6.6±3.9 | 5.1±2.3 | 6.5±2.8 | 6.5±3.2 |

Values are mean ± SD (n = 6). Non significant difference was obtained following one-way ANOVA.
